# Influenza vaccine effectiveness against influenza A subtypes in Europe: Results from the 2021–2022 I‐MOVE primary care multicentre study

**DOI:** 10.1111/irv.13069

**Published:** 2022-11-21

**Authors:** Esther Kissling, Francisco Pozo, Iván Martínez‐Baz, Silke Buda, Ana‐Maria Vilcu, Lisa Domegan, Clara Mazagatos, Frederika Dijkstra, Neus Latorre‐Margalef, Sanja Kurečić Filipović, Ausenda Machado, Mihaela Lazar, Itziar Casado, Ralf Dürrwald, Sylvie van der Werf, Joan O'Donnell, Juan Antonio Linares Dopido, Adam Meijer, Maximilian Riess, Vesna Višekruna Vučina, Ana Paula Rodrigues, Maria Elena Mihai, Jesús Castilla, Luise Goerlitz, Alessandra Falchi, Jeff Connell, Daniel Castrillejo, Mariette Hooiveld, Annasara Carnahan, Maja Ilić, Raquel Guiomar, Alina Ivanciuc, Marine Maurel, Ajibola Omokanye, Marta Valenciano, I‐MOVE study team

**Affiliations:** ^1^ Epiconcept Paris France; ^2^ National Centre for Microbiology Institute of Health Carlos III Madrid Spain; ^3^ Consortium for Biomedical Research in Epidemiology and Public Health (CIBERESP) Madrid Spain; ^4^ Instituto de Salud Pública de Navarra ‐ IdiSNA Pamplona Spain; ^5^ Robert Koch Institut Berlin Germany; ^6^ INSERM, Sorbonne Université Institut Pierre Louis d'épidémiologie et de Santé Publique (IPLESP UMRS 1136) Paris France; ^7^ Health Service Executive‐Health Protection Surveillance Centre Dublin Ireland; ^8^ National Centre for Epidemiology Institute of Health Carlos III Madrid Spain; ^9^ National Institute for Public Health and the Environment (RIVM) Bilthoven The Netherlands; ^10^ The Public Health Agency of Sweden Stockholm Sweden; ^11^ Croatian Institute of Public Health Zagreb Croatia; ^12^ Instituto Nacional de Saúde Dr. Ricardo Jorge Lisbon Portugal; ^13^ “Cantacuzino” National Military Medical Institute for Research and Development Bucharest Romania; ^14^ Unité de Génétique Moléculaire des Virus à ARN, Institut Pasteur Université Paris Cité, UMR 3569 CNRS Paris France; ^15^ CNR virus des infections respiratoires, Institut Pasteur Paris France; ^16^ Subdirección de Epidemiología, Dirección General de Salud Pública, Servicio Extremeño de Salud Extremadura Spain; ^17^ Laboratoire de Virologie Université de Corse‐Inserm Corte France; ^18^ National Virus Reference Laboratory University College Dublin Dublin Ireland; ^19^ Servicio de Epidemiología, DGSP, Consejería de Políticas Sociales, Salud Pública y Bienestar Animal, Ciudad Autónoma de Melilla Melilla Spain; ^20^ Nivel Utrecht The Netherlands; ^21^ European Centre for Disease Prevention and Control Stockholm Sweden

**Keywords:** Europe, influenza, influenza vaccine, multicentre study, vaccine effectiveness

## Abstract

**Background:**

In 2021–2022, influenza A viruses dominated in Europe. The I‐MOVE primary care network conducted a multicentre test‐negative study to measure influenza vaccine effectiveness (VE).

**Methods:**

Primary care practitioners collected information on patients presenting with acute respiratory infection. Cases were influenza A(H3N2) or A(H1N1)pdm09 RT‐PCR positive, and controls were influenza virus negative. We calculated VE using logistic regression, adjusting for study site, age, sex, onset date, and presence of chronic conditions.

**Results:**

Between week 40 2021 and week 20 2022, we included over 11 000 patients of whom 253 and 1595 were positive for influenza A(H1N1)pdm09 and A(H3N2), respectively. Overall VE against influenza A(H1N1)pdm09 was 75% (95% CI: 43–89) and 81% (95% CI: 45–93) among those aged 15–64 years. Overall VE against influenza A(H3N2) was 29% (95% CI: 12–42) and 25% (95% CI: −41 to 61), 33% (95% CI: 14–49), and 26% (95% CI: −22 to 55) among those aged 0–14, 15–64, and over 65 years, respectively. The A(H3N2) VE among the influenza vaccination target group was 20% (95% CI: −6 to 39). All 53 sequenced A(H1N1)pdm09 viruses belonged to clade 6B.1A.5a.1. Among 410 sequenced influenza A(H3N2) viruses, all but eight belonged to clade 3C.2a1b.2a.2.

**Discussion:**

Despite antigenic mismatch between vaccine and circulating strains for influenza A(H3N2) and A(H1N1)pdm09, 2021–2022 VE estimates against circulating influenza A(H1N1)pdm09 were the highest within the I‐MOVE network since the 2009 influenza pandemic. VE against A(H3N2) was lower than A(H1N1)pdm09, but at least one in five individuals vaccinated against influenza were protected against presentation to primary care with laboratory‐confirmed influenza.

## 1 BACKGROUND

Since 2008–2009, the I‐MOVE (Influenza Monitoring Vaccine Effectiveness in Europe) multicentre case control study (MCCS) at primary care level has provided influenza vaccine effectiveness (VE) estimates by influenza virus (sub)type, age group, target population, since 2012–2013 by vaccine type, and since 2015–2016 by genetic clade/variant.^1^, ^2^, ^3^, ^4^, ^5^ The end of 2019 saw the emergence of a novel severe acute respiratory syndrome–coronavirus 2 (SARS‐CoV‐2), which can cause coronavirus disease 2019 (COVID‐19). Many countries implemented mitigation measures against COVID‐19, such as physical distancing, working from home, school closures, mask wearing, and increased use of hand sanitisers. These measures may have also prevented influenza virus transmission. I‐MOVE did not provide influenza VE estimates in 2020–2021 as this season was characterised by the quasi‐absence of influenza virus circulation. Influenza levels in the World Health Organisation (WHO) European regions in the 2021–2022 season were higher, but varied by country.^6^ Influenza virus A(H3N2) dominated in all European countries.^6^ In countries participating in I‐MOVE, influenza vaccination is recommended for older adults (those aged ≥50, ≥55, ≥60, or ≥65 years, depending on the country), among those with medical risk conditions and among children in Ireland.

In February 2021, the WHO recommendations for the influenza A subtypes for the 2021–2022 egg‐based influenza vaccine for the Northern Hemisphere were to include an A/Victoria/2570/2019 (H1N1)pdm09‐like virus (clade 6B.1A.5a.2) and an A/Cambodia/e0826360/2020 (H3N2)‐like virus (clade 3C.2a1b.2a.1). The recommendation for the influenza A(H1N1)pdm09 virus was different for cell‐ or recombinant‐based vaccines, namely, an A/Wisconsin/588/2019 (H1N1)pdm09‐like virus (clade 6B.1A.5a.2).^7^

In this article, we present the I‐MOVE end‐of‐season estimates of 2021–2022 influenza VE among patients presenting with a respiratory infection at primary care level.

## 2 METHODS

The methods of the multicentre case–control study have been described previously and are based on the ECDC generic case–control study protocol and the I‐MOVE+ generic study protocol.^1^, ^5^, ^8^, ^9^, ^10^

Briefly, study sites in nine European countries took part in the primary care‐based I‐MOVE multicentre study in the 2021–2022 influenza season: Croatia, France, Germany, Republic of Ireland, the Netherlands, Portugal, Romania, Spain (contributing with two distinct study sites), and Sweden. General practitioners (GPs), or paediatricians, systematically selected patients, or selected all patients presenting with influenza‐like illness (ILI) or acute respiratory infection (ARI) to include in the study. Physicians either sampled patients or referred them to a medical laboratory or a COVID‐19 testing centre. Physicians obtained information on patients using interviews and linkage to electronic health records. All study sites collected the symptoms, date of onset and swabbing, 2021–2022 influenza vaccination status and date of vaccination, sex, age, presence of chronic conditions, and influenza and SARS‐CoV‐2 test results.

In the pooled analysis, we included patients with a specimen taken less than 8 days after symptom onset. Using the test‐negative design, a case of confirmed influenza was an ARI or ILI patient who was sampled and tested positive for influenza A(H1N1)pdm09 or A(H3N2) virus (for an influenza A subtype‐specific analysis) using real‐time reverse‐transcription polymerase chain reaction (RT‐PCR).^11^ Controls were ARI/ILI patients who tested negative for any influenza virus.

For each study site, we included patients presenting symptoms 14 or more days after the start of national influenza vaccination campaigns. In each study site, controls were excluded if presenting in weeks of onset prior to the first influenza (sub)type positive case for each (sub)type‐specific analysis.

For one study site not collecting date of symptom onset, we imputed it as 2 days before the sample date, as 2 days was the median delay between onset and sample in the pooled data.

We defined a person as vaccinated if he or she had received 2021–2022 influenza vaccine 14 or more days before symptom onset. Patients vaccinated fewer than 14 days before symptom onset were excluded.

One of the Spanish study sites (Navarra) is a comprehensive surveillance system, where patients from all GPs in the region are included, compared with the sentinel system of other study sites. We included all cases, but only a random sample of 20% of controls by onset week for Navarra, to account for the differences in absolute numbers between the systems.

We excluded from the pooled analysis any study site that had less than 10 influenza (sub)type‐specific cases for each (sub)type‐specific analysis. We combined individual patient data and used a one‐stage model, with study site as a fixed effect. We carried out a complete case analysis and used a logistic regression model to calculate VE including potential confounding factors: Age (modelled as a restricted cubic spline with four or five knots, age groups or age as a linear term depending on the analysis, as determined by the Akaike Information criterion [AIC]), sex, presence of at least one chronic condition (including pregnancy and obesity where available and applicable), and date of onset (modelled as a restricted cubic spline with four or five knots, depending on the AIC).

We stratified the data into patients aged 0–14 years, 15–64 years, and those aged 65 years and older to obtain age group‐specific VE. We estimated VE among the target group for influenza vaccination. We also estimated VE by time since vaccination, comparing unvaccinated to those vaccinated <90, 90–119, 120–149, and ≥150 days before symptom onset.

In a sensitivity analysis, we dropped COVID‐19 positive controls,^12^ as a correlation between influenza and COVID‐19 vaccination could potentially bias VE estimates, by violating a fundamental criterion of the test‐negative design: Controls are not affected by the vaccination under study.^11^

In nine study sites, all or a random sample of influenza viruses were selected for sequencing (haemagglutinin genome segment and/or whole genome). In one study site (France), only samples going to one of three labs used within the study carried out sequencing. Haemagglutinin (HA) sequences were uploaded by each site to the GISAID Epiflu database and downloaded for centralised phylogenetic and amino acid substitution analysis of the HA1 coding portion in MEGA6 to determine clade distribution, at the National Influenza Centre, Madrid.

## 3 RESULTS

### 3.1 Participant profiles

**FIGURE 1:**
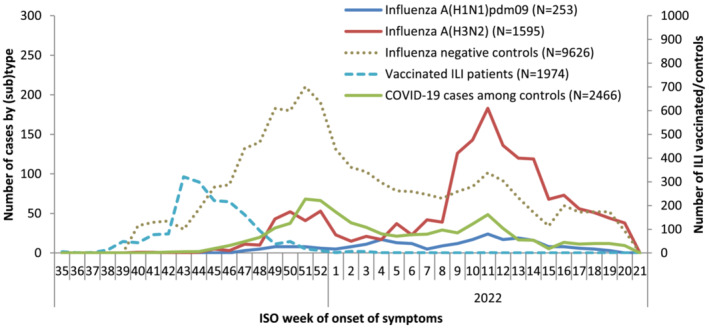
Number of ILI/ARI reports by case status, week of symptom onset, and number of SARS‐CoV‐2 cases among influenza‐negative controls, I‐MOVE primary care multicentre case control study, 2021–2022 influenza season

We excluded 961 patients (7.6%) from the complete case analysis, due to missing data for age, sex, chronic condition or influenza vaccination status, including one study site with too few influenza cases for analysis (<10 influenza positive cases). We included 253 influenza A(H1N1)pdm09‐ and 1595 influenza A(H3N2)‐positive cases and 9626 influenza‐negative controls between ISO weeks 40 2021 and 20 2022 (Figure 1). Only France, Germany, and the Netherlands had a sufficient sample size (10 or more cases) to be included in the A(H1N1)pdm09 analyses.

The proportion vaccinated with the 2021–2022 influenza vaccine was 18% among controls (Table 1). Among vaccinated controls, vaccine brand was known for 1328 of 1731 (77%) patients, and almost all (1322; 99.5%) had received egg‐propagated vaccines. Of the 1328 controls with vaccine brand documented, 850 (64%) received a non‐adjuvanted normal‐dose egg‐propagated inactivated quadrivalent vaccine, 395 (30%) received an adjuvanted vaccine, 49 (4%) received a high dose quadrivalent vaccine, 28 (2%) a live attenuated influenza vaccine, and six (<1%) a quadrivalent cell‐based vaccine.

**TABLE 1:** Characteristics of influenza A(H1N1)pdm09 (*n* = 253), A(H3N2) (*n* = 1595), and controls (*n* = 9626) included in the I‐MOVE primary care multicentre case control study, influenza season 2021–2022

Variables	Number of influenza A(H1N1)pdm09 cases/total *n*(%) *N* = 253	Number of influenza A(H3N2) cases/total *n*(%) *N* = 1595	Number of test‐negative controls/total *n*(%) *N* = 9626
**Median age**	27	24	33
**Age groups**			
0–4	55/253 (22)	130/1595 (8)	1784/9626 (19)
5–14	53/253 (21)	301/1595 (19)	1212/9626 (13)
15–64	136/253 (54)	1002/1595 (63)	5612/9626 (58)
≥65	9/253 (4)	162/1595 (10)	1018/9626 (11)
**Sex**			
Female	125/253 (49)	830/1595 (52)	4713/9626 (49)
**Days between onset of symptoms and swabbing**			
0	13/253 (5)	38/1595 (2)	300/9626 (3)
1	91/253 (36)	259/1595 (16)	1568/9626 (16)
2	65/253 (26)	830/1595 (52)	5062/9626 (53)
3	47/253 (19)	231/1595 (14)	1155/9626 (12)
4–7	37/253 (15)	237/1595 (15)	1541/9626 (16)
**Seasonal influenza vaccination, 2021–**20**22**	7/253 (3)	227/1595 (14)	1731/9626 (18)
**Current and previous season influenza vaccination status**			
Not vaccinated	205/213 (96)	980/1186 (83)	5910/7619 (78)
Current season vaccination only	0/213 (0)	23/1186 (2)	201/7619 (3)
Previous season vaccination only	4/213 (2)	55/1186 (5)	496/7619 (7)
Current and previous season vaccination	4/213 (2)	128/1186 (11)	1012/7619 (13)
Missing	40	409	2007
**Seasonal vaccination types** ^a^			
Quadrivalent vaccine^b^	5 (100)	80 (51)	850 (64)
Adjuvanted vaccine	0 (0)	74 (47)	395 (30)
High dose quadrivalent	0 (0)	0 (0)	49 (4)
LAIV	0 (0)	2 (1)	28 (2)
Quadrivalent cell‐based	0 (0)	1 (1)	6 (<1)
Unknown	2	70	403
**≥1 chronic condition**	36/253 (14)	358/1595 (22)	2281/9626 (24)
**Belongs to target group for vaccination**	41/253 (16)	445/1595 (28)	3090/9624 (32)
Missing	0	0	2
**SARS‐CoV‐2 positive**	5/253 (2)	41/1595 (3)	2466/9625 (26)
Missing	0	0	1

Abbreviation: LAIV, live attenuated influenza vaccine.

^a^ Among vaccinated.

^b^ Quadrivalent, normal dose, inactivated, non‐adjuvanted egg‐propagated.

The median age was 33 years among controls and 27 and 24 years among influenza A(H1N1)pdm09 and A(H3N2) cases, respectively (Table 1). Among controls 19% were aged 0–4 years old compared with 22% and 8% among influenza A(H1N1)pdm09 and A(H3N2) cases, respectively.

### 3.2 Genetic characterisation

Among study sites included in the pooled analysis, eight study sites systematically sequenced influenza positive specimens or sequenced all specimens technically possible. Among the 153 influenza A(H1N1)pdm09 cases included in the analysis from those countries/labs sequencing viruses, and part of the A(H1N1)pdm09 analysis (DE, FR, NL), 53 (35%) were sequenced (Table 2). Of the 53 sequenced, all belonged to clade 6B.1A.5a.1, represented by A*/Guangdong‐*Maonan/SWL1536/2019. No viruses belonged to clade 6B.1A.5a.2, the vaccine virus clade.

**TABLE 2:** Genetic group distribution of viruses sequenced among study sites participating in the random sequencing of influenza positive specimens. I‐MOVE primary care multicentre case control study, influenza season 2021–2022

	Clade	*n*	%
**Total influenza A(H1N1)pdm09** ^a^		***n* = 153**	
Sequenced^b^		53	*35*
A/*Guangdong‐*Maonan/SWL1536/2019‐like	6B.1A.5a.1	53	100
**Total influenza A(H3N2)** ^a^		***N* = 1354**	
Sequenced^b^		410	*30*
A*/Bangladesh*/4005/2020‐like	3C.2a1b.2a.2	402	98
	No subgroup	1	<1
	New subgroup^c^	63	15
	Subgroup (i)	1	<1
	Subgroup (iii)	64	16
	Subgroup (iv)	273	68
A/*Denmark*/3264/2019	3C.2a1b.1a	8	2

^a^ Among study sites and labs providing data from sequencing (countries included: DE, ES, FR, IE, NA, NL, PT, SE; two labs in FR not included).

^b^ Among patients included in the pooled analysis after restrictions.

^c^ Harbouring E50K, F79V, I140K mutations.

Among the 1354 influenza A(H3N2) cases included in the I‐MOVE analysis from the countries/labs providing sequencing information, 410 (30%) were sequenced (Table 2). Four hundred and two viruses (98%) belonged to clade 3C.2a1b.2a.2, represented by A/*Bangladesh*/4005/2020‐like and eight viruses (2%) belonged to clade 3C.2a1b.1a represented by A/*Denmark*/3264/2019. Among the 402 viruses belonging to clade 3C.2a1b.2a.2, one (<1%) belonged to subgroup (i), harbouring S205F and A212T mutations; 64 (16%) belonged to subgroup (iii), harbouring D53N mutations; and 273 (68%) belonged to subgroup (iv), harbouring D53G mutations. Sixty‐four clade 3C.2a1b.2a.2 viruses (16%) did not belong to a designated 3C.2a1b.2a.2 subgroup, with one harbouring no further important mutations and 63 harbouring E50K, F79V and I140K mutations characterising a new emerging cluster. No viruses belonged to the 3C.2a1b.2a.1 clade, the vaccine virus clade.

### 3.3 Vaccine effectiveness estimates

The overall adjusted VE against influenza A(H1N1)pdm09 was 75% (95% CI: 43–89) (Table 3). The adjusted VE against influenza A(H1N1)pdm09 was 81% (95% CI: 45–93) among 15–64 year olds. Sample size did not allow VE estimation among other age strata.

The overall adjusted VE against influenza A(H3N2) was 29% (95% CI: 12–42) (Table 3). The adjusted VE against influenza A(H3N2) was 25% (95% CI: −41 to 61) among 0‐ to 14‐year‐olds, 33% (95% CI: 14–49) among 15‐ to 64‐year‐olds, and 26% (95% CI: −22 to 55) among those aged 65 and older. VE within the target group for vaccination was 20% (95% CI: −6 to 39). The adjusted VE against influenza A(H3N2) among those without presence of chronic condition was 34% (95% CI: 13–50) and 21% (95% CI: −8 to 43) among those with presence of a chronic condition.

**TABLE 3:** Pooled seasonal vaccine effectiveness against any influenza, A(H1N1)pdm09 and A(H3N2), overall, by age groups and target group for vaccination. I‐MOVE primary care multicentre case control study, influenza season 2021–2022

Influenza (sub)type	Age group	Population	*N* ^a^	Cases;vacc/controls; vacc	Adjusted VE	95% CI
Any influenza	All ages		11 643	2017;260/9626;1731	33	20–45
	0–14 years		3562	566;17/2996;133	28	−30 to 60
	15–64 years		6876	1264;107/5612;873	41	25–54
	65+ years		1205	187;136/1018;725	23	−21 to 51
	All ages	Target group	3630	540;211/3090;1257	23	0–40
	All ages	No chronic condition	8923	1578;105/7345;820	37	18–51
	All ages	Chronic condition	2720	439;155/2281;911	28	3–46
A(H1N1)pdm09^b^	All ages		2570	253;7/2317;261	75	43–89
	15–64 years		1320	136;4/1184;135	81	45–93
A(H3N2)	All ages		11 201	1595;227/9606;1724	29	12–42
	0–14 years		3422	431;14/2991;133	25	−41 to 61
	15–64 years		6600	1002;93/5598;867	33	14–49
	65+ years		1141	162;120/979;698	26	−22 to 55
	All ages	Target group	3528	445;186/3083;1255	20	−6 to 39
	All ages	No chronic condition	8569	1237;91/7332;815	34	13–50
	All ages	Chronic condition	2632	358;136/2274;909	21	−8 to 43

Abbreviations: CI, 95% confidence interval; VE, vaccine effectiveness.

^a^ Based on the complete case analysis: records missing values for age or sex or chronic condition or vaccination status are dropped.

^b^ Due to low sample size, VE estimation in all strata is not performed.

**FIGURE 2:**
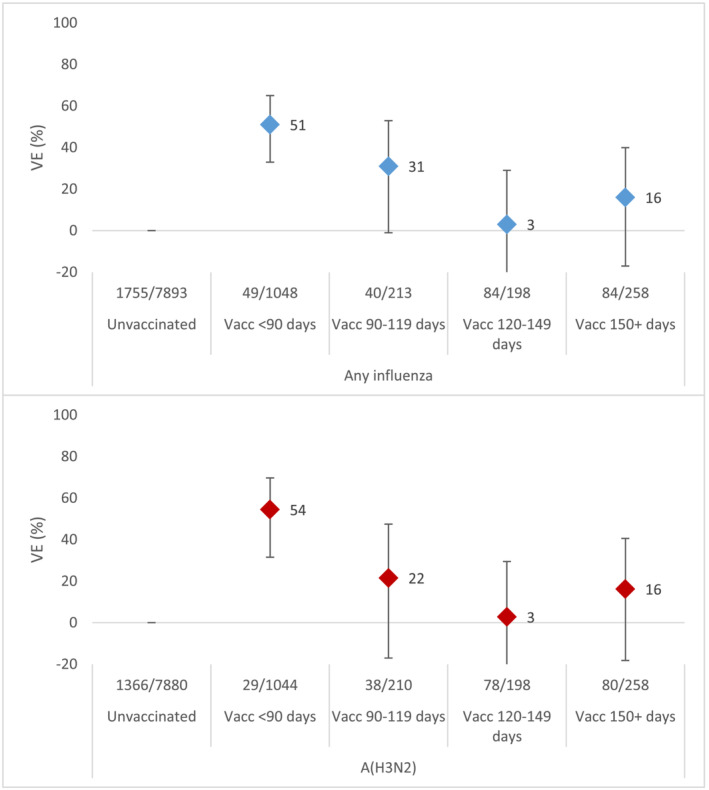
All influenza (upper figure, in blue) and influenza A(H3N2) (lower figure, in red) vaccine effectiveness by days since vaccination. I‐MOVE primary care multicentre case control study, influenza season 2021–2022

We dropped 20 patients (<1%) with inexact influenza vaccination date for the analysis by time since influenza vaccination. The VE against influenza A(H3N2) among all ages was 54% (95% CI: 32–70) among those presenting with symptoms <90 days, 22% (95% CI: −17 to 47) among those presenting 90–119 days, 3% (95% CI: −34 to 29) among those presenting 120–149 days, and 16% (95% CI: −18 to 41) among those presenting ≥150 days since vaccination (Figure 2).

### 3.4 Sensitivity analyses: Excluding SARS‐CoV‐2 positive controls

When excluding SARS‐CoV‐2 positive patients among controls, the overall adjusted VE against influenza A(H1N1)pdm09 was 79% (95% CI: 52–91) and 83% (95% CI: 50–94) among 15–64 year olds (Table 4).

**TABLE 4:** Pooled seasonal vaccine effectiveness against influenza A(H1N1)pdm09 and A(H3N2), overall and by age groups excluding SARS‐Cov‐2 positive patients among controls. I‐MOVE primary care multicentre case control study, influenza season 2021–2021

Influenza (sub)type	Age group	Population	*N* ^a^	Cases;vacc/controls; vacc	Adjusted VE	95% CI
A(H1N1)pdm09	All ages		2067	253;7/1814;202	79	52–91
	15–64 years		1003	136;4/867;104	83	50–94
A(H3N2)	All ages		8743	1595;227/7148;1250	36	21–49
	0–14 years		3043	431;14/2612;118	35	−24 to 66
	15–64 years		4795	1002;93/3793;587	39	19–54
	65+ years		873	162;120/711;522	31	−18 to 60
	All ages	Target group	2672	445;186/2227;931	28	4–47

Abbreviations: CI, 95% confidence interval; VE, vaccine effectiveness.

^a^ Based on the complete case analysis: records missing values for age or sex or chronic condition or vaccination status are dropped.

The overall adjusted VE against influenza A(H3N2) when excluding SARS‐CoV‐2 controls was 36% (95% CI: 21–49) (Table 4). The adjusted VE against influenza A(H3N2) was 35% (95% CI: −24–66) among 0‐ to 14‐year‐olds, 39% (95% CI: 19–54) among 15‐ to 64‐year‐olds, and 31% (95% CI: −18 to 60) among those aged 65 and older. VE within the target group for vaccination was 28% (95% CI: 4–47).

## 4 DISCUSSION

In the 2021–2022 influenza season, there was overall circulation of influenza A viruses in communities of the study sites in the I‐MOVE primary care network, with a predominance of influenza A(H3N2), and only three study sites could be included in the VE analysis against A(H1N1)pdm09.

VE against influenza A(H1N1)pdm09 was 75% among all ages and 81% amongst those aged 15–64 years. Sample size did not permit VE estimates in any other strata/groups. The VE against influenza A(H3N2) was 29% among all ages, with similar estimates (ranging between 25% and 33%) by age group. Within the target group for vaccination, VE was 20% against A(H3N2). VE against influenza A(H3N2) was 21% among those with a chronic condition and 34% among those without a chronic condition.

The 2021–2022 VE point estimates against A(H1N1)pdm09 among all ages was higher than previously estimated within the I‐MOVE primary care network in the post 2009 A(H1N1)pdm09 pandemic seasons^2^, ^13^, ^14^; however, in the 2021–2022 study, few study sites/cases were included. The A(H1N1)pdm09 vaccine component this season was clade 6B.1A.5a.2, different from the circulating clade 6B.1A.5a.1. This circulating virus did not show good reactivity with ferret antiserum raised against the vaccine virus,^15^ which we would not expect with a high VE. There are some suggestions that ferret models may not be the most appropriate model for antigenic changes in humans.^16^, ^17^ Indeed, this high VE was also observed in a 2021–2022 UK study of A(H1N1)pdm09 VE against influenza in those requiring emergency department visits (75%; 95% CI: 30–92).^18^ Whereas ferrets are naïve, humans may have experienced previous influenza infection and may have residual effects of vaccination. Indeed, the 2020–2021 Northern Hemisphere influenza A(H1N1)pdm09 component was 5B.1A.5a.1, although more research is needed to fully understand the high VE.

The VE point estimate against A(H3N2) among all ages was higher at 29% than the overall I‐MOVE A(H3N2) VE in the 2017–2018 and 2018–2019 seasons, where VE ranged from −1% to 13%, similar to that in the 2016–2017 season (28%) and lower than in the 2019–2020 season at 49%.^13^, ^19^ The VE was also higher than the interim VE reported in the United States (16%) but similar to that in Denmark against influenza A (25% among those aged 7–44 years) and in the aforementioned UK study (28%).^18^, ^20^, ^21^ The majority (98%) of the viruses sequenced in the 2021–2022 season belonged to clade 3C.2a1b.2a.2. Ferret antiserum raised against the 3C.2a1b.2a.1 vaccine virus recognised the circulating 3C.2a1b.2a.2 virus poorly.

The A(H3N2) VE declined with more time since vaccination at 54% among those vaccinated <90 days before symptom onset and 16% among those vaccinated 150 days or more before symptom onset. This waning of the vaccine effect with time since vaccination was observed in other long and late seasons in the I‐MOVE primary care network.^22^ Waning of the vaccine effect against A(H3N2) has been observed in other seasons and elsewhere^23^, ^24^, ^25^; however, other studies also suggest a protection against current season A(H3N2) viruses from previous season vaccines.^26^, ^27^ Not all sites in our study collected information on prior season influenza vaccination, but where known, the VE of previous season influenza vaccination (without current season vaccination) was 14% (95% CI: −20 to 38), compared with those vaccinated in neither season (data not shown). The waning of the vaccine effect and the potential protection of an individual by previous season vaccination and/or previous season infection needs to be further studied by age group and in light of circulating genetic variants in other seasons where sample size allows.

VE against A(H3N2) was lower among the target group for vaccination (20%) and also lower among those with presence of at least one chronic condition (21%) compared with all ages (29%). It may be that changing guidelines around personal protective behaviour related to the COVID‐19 pandemic meant that persons at risk were less likely to be exposed to the influenza virus early in the season. More relaxed COVID‐19 guidance later in the season may have meant people in risk groups were more likely to be influenza cases later in the season when the influenza vaccine effect may have declined. Those belonging to the influenza vaccine target group were more likely to have presented 120 days or more since vaccination compared with those not in the target group (35% vs. 24%, respectively; *P* < .001). VE against A(H3N2) was lower among the target group for vaccination in the 2019–2020 season (49% vs. 32%; unpublished data). However, it may be that individuals in risk groups respond less well to vaccination, due to suboptimal immune responses.

Study sites included in this multicentre study faced challenges around maintaining influenza primary care sentinel systems due to changes in swabbing guidance, health‐care‐seeking guidance, and several different patient pathways for diagnosis of patients with respiratory illness.^28^ It is important to critically assess if these changes to health‐care seeking guidance, behaviour, and structures brought on by the COVID‐19 pandemic may have introduced change in our VE estimates compared with pre‐pandemic times or could have introduced any biases in our estimates. Given the antigenic information, the A(H1N1)pdm09 VE estimates may be higher than expected. If this is indeed a bias, then we would also expect the A(H3N2) to be biased upwards. However, A(H3N2) VE estimates are compatible with what we may expect. Although haemagglutination inhibition (HAI) assays do not always correlate well with VE, the changes to surveillance systems and to human behaviour brought about by the COVID‐19 pandemic may have modified the VE in certain ways. This could have included a selection bias in surveillance systems that have been subject to change due to changes in health‐care seeking guidance during the pandemic or differential mixing between vaccinated and unvaccinated. These potential biases are difficult to assess within the test‐negative design. A further bias we investigated in a sensitivity analysis was that of inclusion of SARS‐CoV‐2 patients among controls. With a high correlation of influenza and COVID‐19 vaccination, the control group (if SARS‐CoV‐2 controls were included) may be influenced by the vaccination, thus violating one of the key test‐negative design assumptions.^12^ When excluding SARS‐CoV‐2 controls, the VE against A(H1N1)pdm09 was ≤4% and against A(H3N2) ≤9% higher for all estimates in absolute percentages. More research is needed to understand if there are any effects of excluding a large proportion of controls in the context of high incidence of COVID‐19.

Despite potential limitations, this study contributes to the 2021–2022 influenza VE results, of which only few were published at time of writing in peer‐reviewed journals.^20^, ^21^, ^27^, ^29^ This multicentre study, including influenza A subtype‐specific VE has a sample size high enough for stratified estimates and presents sequencing results.

## 5 CONCLUSIONS

Results from the I‐MOVE network indicated high overall VE against influenza A(H1N1)pdm09, with lower VE against influenza A(H3N2) presentation in primary care in the 2021–2022 influenza season. In this season, at least one in five individuals vaccinated against influenza were protected against presentation in primary care with laboratory‐confirmed influenza. Careful assessment of bias in light of changing structures and guidelines at primary care level should be made.

## CONFLICT OF INTEREST

All authors declare no conflict of interest.

## AUTHOR CONTRIBUTION

Esther Kissling was involved in the original methodological design of the study (generic protocol). She contributed to the coordination of the I‐MOVE network and undertook the statistical analysis on which the research article is based. She led the manuscript writing, interpreted results, and approved the final version of the manuscript. Francisco Pozo coordinated the I‐MOVE virological analysis of the primary care study, helped interpret results, and read, contributed to, and approved the final version of the manuscript. Iván Martínez‐Baz, Silke Buda, Ana‐Maria Vilcu, Lisa Domegan, Clara Mazagatos, Frederika Dijkstra, Neus Latorre‐Margalef, Sanja Kurečić Filipović, Ausenda Machado, Mihaela Lazar, Itziar Casado, Ralf Dürrwald, Sylvie van der Werf, Joan O'Donnell, Juan Antonio Linares Dopido, Adam Meijer, Maximilian Riess, Vesna Višekruna Vučina, Ana Paula Rodrigues, Maria Elena Mihai, Jesús Castilla, Luise Goerlitz, Alessandra Falchi, Jeff Connell, Daniel Castrillejo, Mariette Hooiveld, Annasara Carnahan, Maja Ilić, Raquel Guiomar, and Alina Ivanciuc were responsible for the coordination of the study at the national/regional level and contributed to developing the study site specific protocols. They were in charge of the data collection and management and validating the clinical and laboratory data published in this research article. They interpreted the results and read, contributed to, and approved the final version of the manuscript. Marine Maurel was involved in the statistical analysis on which the research article is based. She reviewed the manuscript and approved the final version of the manuscript. Ajibola Omokanye was involved in study design, interpretation of results, review of the manuscript, and approval of the final version of the manuscript. Marta Valenciano initiated the original methodological design of the study. She was involved in the coordination of I‐MOVE network, interpretation of results, contributed to manuscript writing, and approved the final version of the manuscript. The I‐MOVE study team contributors contributed to developing the study‐site specific protocols at primary care sites at national/regional level. They were in charge of supervising the study and collecting and validating the clinical and laboratory data published in this research article. They read, contributed to, and approved the final version of the manuscript.

## ETHICS STATEMENT

The planning, conduct and reporting of the studies was in line with the Declaration of Helsinki. Official ethical approval and patient consent was not required the Netherlands and Spain, as these studies were classified as being part of routine care/surveillance. Other study sites obtained local ethical approval from a national review board, according to local site regulations, as follows: Croatia: approved by the Ethics Committee of the Croatian Institute of Public Health (class 030‐02/21‐01/8); France: 471393; Germany: EA2/126/11; Ireland: ICGP2019.4.04; Portugal: approved 18 January 2012 by the Ethics Committee of Instituto Nacional de Saúde Doutor Ricardo Jorge, no registration number given; Romania: CE354/30.09.2019; Sweden: 2006/1040–31/2.

### PEER REVIEW

The peer review history for this article is available at https://publons.com/publon/10.1111/irv.13069.

## Data Availability

Data available only on request due to privacy/ethical restrictions: The data that support the findings of this study may be available on request from the corresponding author, depending on the request. The data are not publicly available due to privacy or ethical restrictions.
